# A Unique Presentation of Spontaneous Pneumomediastinum Following COVID-19 Infection

**DOI:** 10.7759/cureus.24565

**Published:** 2022-04-28

**Authors:** Ravi Soni, William Brewster, Woodwin Weeks, Steven Graves

**Affiliations:** 1 Osteopathic Medicine, Philadelphia College of Osteopathic Medicine, Moultrie, USA; 2 Department of Family and Community Medicine, Colquitt Regional Medical Center, Moultrie, USA; 3 Department of Emergency Medicine, Colquitt Regional Medical Center, Moultrie, USA

**Keywords:** follow-up appointment, pneumothorax (ptx), subcutaneous emphysema, covid 19, spontaneous pneumomediastinum (spm)

## Abstract

Pneumomediastinum is a rare, life-threatening condition in which air leaks into the mediastinum. Usually, it results from a traumatic event that leads to the escape of air from the airway, lungs, or bowel into the chest cavity. Patients with underlying lung pathology or a history of invasive mechanical ventilation have an increased risk of developing a pneumomediastinum. A spontaneous pneumomediastinum (SPM) occurs in the absence of these risk factors. Patients with coronavirus disease 2019 (COVID-19) pneumonia tend to have a higher risk of developing an SPM, however, this is usually linked to mechanical ventilator use. Although rare, cases of healthy young patients with no history of underlying lung pathology or mechanical ventilator use developing an SPM are increasingly being reported. In efforts to bring more attention to this complication, we present the case of an SPM in a 40-year-old female patient with COVID-19 pneumonia and highlight the importance of close follow-up.

## Introduction

Pneumomediastinum is defined as the presence of free air between the structures of the mediastinum. It can be classified as primary, secondary, or iatrogenic. Primary causes, usually rupture of pulmonary blebs, lead to the development of spontaneous pneumomediastinum (SPM). Secondary causes include traumatic injuries such as penetrating trauma to the chest or abdominal wall and nontraumatic causes including conditions that can lead to a rupture in the tracheobronchial tree or the esophagus. Underlying conditions such as asthma, chronic obstructive pulmonary disease (COPD), interstitial lung disease, lung cancers, and chronic alcohol use can increase the risk of a secondary cause of pneumomediastinum. Iatrogenic causes are related to procedures such as endoscopy, mechanical intubation, or central venous line placement [1].

SPM is the presence of free air or gas in the mediastinum that is not associated with traumatic or iatrogenic etiology. In the case of coronavirus disease 2019 (COVID-19), cases of pneumomediastinum are mostly iatrogenic in nature (i.e., mechanical ventilation). Limited cases have been reported in healthy patients with no underlying lung disease or history of tobacco use who develop an SPM in the absence of mechanical ventilation [1,2]. In this article, we present the case of an SPM occurring in a young, healthy female with no underlying lung disease or history of tobacco use who presented to our clinic for a follow-up evaluation after a recent hospitalization for COVID-19 pneumonia.

## Case presentation

A 40-year-old female patient with no significant medical history presented to the family medicine clinic for a scheduled follow-up visit three days after hospital discharge. The patient had presented to the local emergency department six days prior for evaluation of fatigue, weakness, dyspnea, and hypoxia. She was admitted and treated for acute respiratory failure, hypoxemia, and bilateral pneumonia secondary to COVID-19 infection. Her inpatient treatment regimen included remdesivir, baricitinib, dexamethasone, azithromycin, and supplemental oxygen. On day three, she was stabilized and transitioned to outpatient treatment with oral dexamethasone, guaifenesin, albuterol, and supplemental oxygen as needed. She noted an improvement in her symptoms after her discharge but continued to be dyspneic and hypoxic at home with oxygen saturations in the mid to high 80s. Upon arrival at the family medicine clinic, her only complaint was her dyspnea, but she otherwise felt better. Physical exam showed no significant rales, rhonchi, or wheezing, mild tachycardia with no murmurs, and no lower or upper extremity edema was appreciated. Her pulse oximetry reading in the office was 83% on room air. She did not have her supplemental oxygen with her at the office. Given the patient’s improved symptomatology and no remarkable finding on the physical exam aside from the hypoxia, the patient was instructed to continue her current treatment plan at home with dexamethasone, albuterol, guaifenesin, and supplemental oxygen with strict follow-up instructions.

Four days later, she returned to the clinic complaining of worsening dyspnea, as well as a painful and edematous neck. Her physical exam showed hypoxia with an oxygen saturation of 83% on room air, conversational dyspnea, coarse breath sounds bilaterally, tachycardia, and subcutaneous emphysema throughout her neck and upper chest wall. Given her worsening condition and the presence of the subcutaneous emphysema, she was transported to the emergency department for emergent evaluation.

**Figure 1:**
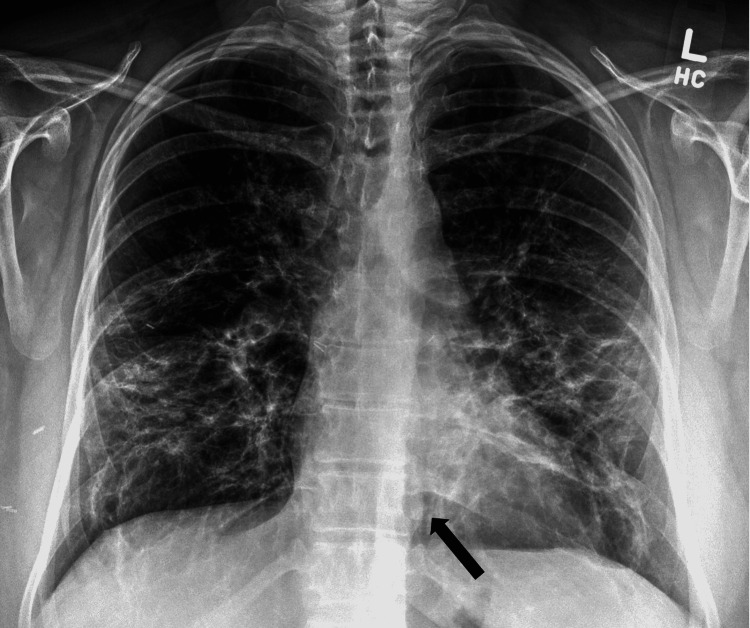
Chest X-ray showing worsening of the infiltrates with pneumomediastinum (see arrow) and subcutaneous emphysema

**Figure 2:**
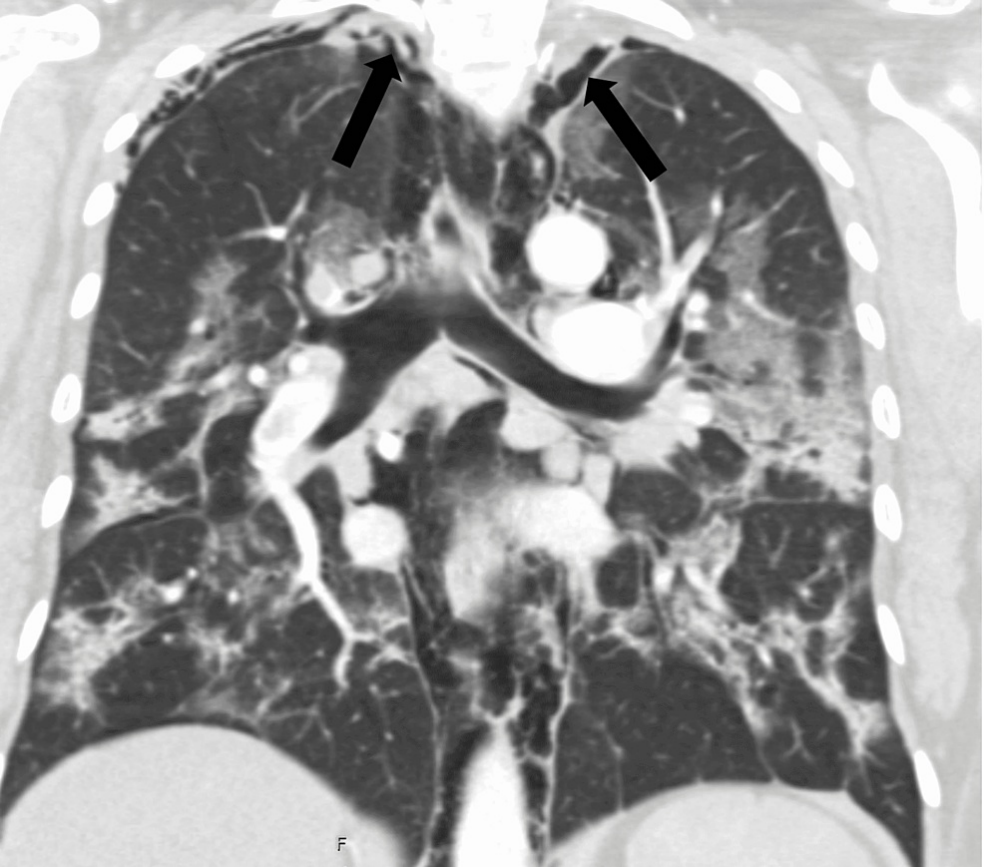
Pneumothroaces at the apex seen here with the pneumomediastinum and bilateral infiltrates

**Figure 3:**
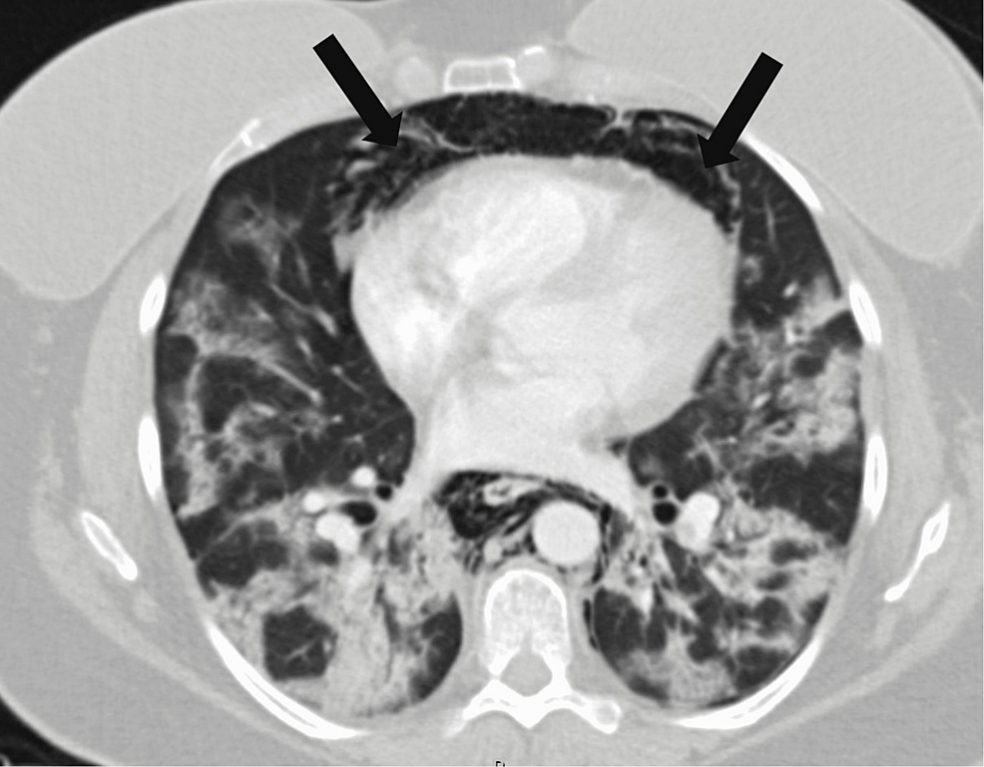
Axial view of pneumomediastinum

**Figure 4:**
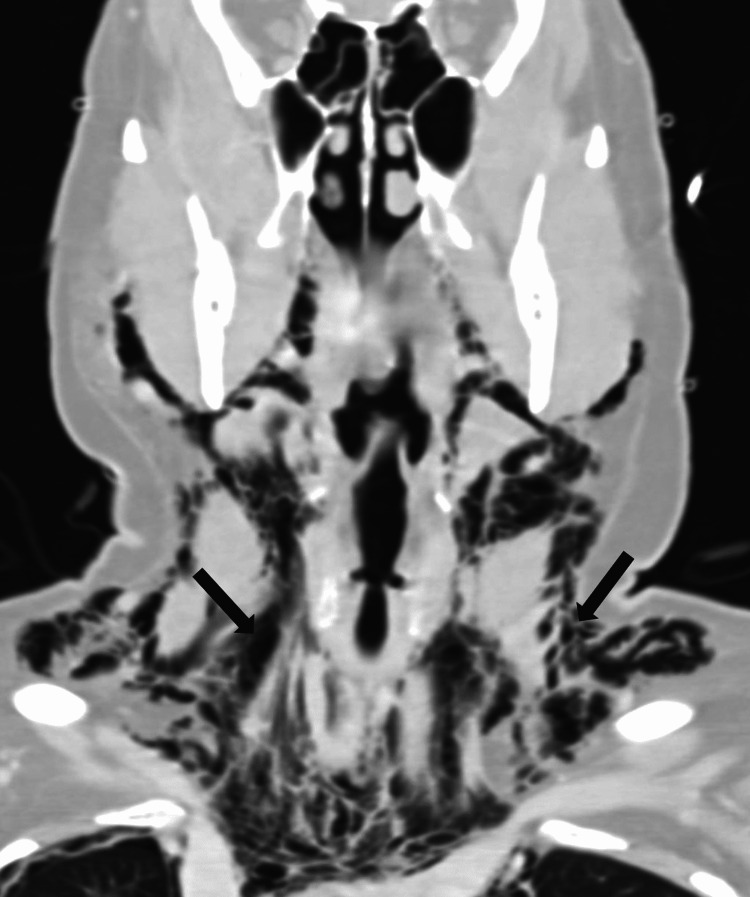
Neck CT showing extensive subcutaneous emphysema in the soft tissues of the neck

Vital signs on arrival to the emergency department were notable for hypoxia with oxygen saturation of 83% on room air and tachycardia (Table 1). She immediately required 5 L of supplemental oxygen to maintain saturations above 90%. A chest X-ray and CT of the neck and thorax were taken which revealed extensive bilateral pulmonary emboli, extensive bilateral infiltrates (Figure 1), pneumothoraces over the apex of the lungs (Figure 2), and pneumomediastinum extending from the thoracic inlet to the skull base without evidence of esophageal rupture (Figures 3-4). Laboratory studies were significant for an elevated white blood cell count of 15.3 thousand/MM^3^, platelet count of 540 thousand/MM^3^, aspartate transaminase (AST) and alanine aminotransferase (ALT) values of 81 U/L and 195 U/L, respectively, lactic acid of 1.65 mmol/L, procalcitonin of 0.08 ng/mL, and a D-Dimer of 9.18 mg/L. EKG was notable for sinus tachycardia with a ventricular rate of 121 beats per minute without significant T wave or ST-segment changes.

**Table 1:** Vital signs

Vital sign	Result
Temperature	98.4°F
Pulse rate	134 beats/min
Respiratory rate	24 breaths/min
Blood pressure	162/110 mmHg
O_2_ saturation by pulse oximetry	83%

She was admitted to the inpatient service with cardiology, pulmonology, and ear, nose, and throat (ENT) consultations. She was started on a heparin infusion, vancomycin, meropenem, clindamycin, methylprednisolone, and diphenhydramine. She underwent bilateral pulmonary artery catheterization, which showed a mean pulmonary arterial pressure of 23 mmHg. Given a normal pulmonary arterial pressure and a normal echocardiogram with no signs of right heart strain, she did not meet the criteria for a thrombectomy. The subcutaneous emphysema and the pneumomediastinum did not require any urgent or invasive intervention. She was closely monitored with daily chest X-rays which showed improvement each day. On hospital day eight, the pneumomediastinum and subcutaneous emphysema had significantly regressed, and her dyspnea had improved. Given her improvement, she was discharged with supplemental oxygen, amoxicillin/clavulanate potassium, benzonatate, and apixaban with instructions for close follow-up. During her 1-week follow-up evaluations in the clinic, her respiratory status continued to improve, and her chest X-ray (CXR) showed resolution of the pneumomediastinum with decreased pulmonary infiltrates. At her 3-month follow-up, she was off supplemental oxygen altogether and returned to her baseline function without complications.

## Discussion

SPM is a rare complication that is showing increased incidence in patients suffering from severe acute respiratory distress syndrome (ARDS) secondary to COVID-19 pneumonia. The proposed mechanism of injury seems to be multifactorial, with diffuse alveolar damage and a pressure imbalance between the alveoli and interstitium being the main factors [2]. Diffuse alveolar damage is caused by a combination of the cytokines released by the severe acute respiratory syndrome coronavirus (SARS-CoV) and the pro-inflammatory cells that respond to these cytokines, leading to alveolar tissue damage [3]. The weakened alveoli are unable to handle the increased intra-alveolar pressure caused by the frequency of coughing and deep breathing in these patients. As a result, the alveoli can rupture and the air can leak into the pulmonary interstitium, traveling along the bronchovascular sheaths and toward the mediastinum, a phenomenon known as the Macklin effect [4-5]. Fortunately, most cases of pneumomediastinum can be managed conservatively with supportive care and close monitoring as the tissues eventually reabsorb the air [1,4].

Though most cases of SPM are self-limited and managed with conservative treatment, life-threatening pathology must be ruled out, and the patient must be monitored closely. This case highlights the importance of close follow-up in patients recovering from hospitalization for COVID-19 pneumonia, as the disease process can quickly progress to a life-threatening emergency such as cardiac tamponade or tracheal obstruction [1,6]. In this case, the patient’s secondary worsening after an initial improvement in symptomatology warranted emergent evaluation and treatment.

Although it seems rare, further research into the current literature shows increasing reports of SPM seen in patients with COVID-19 pneumonia who did not require mechanical ventilation [2,7]. One common factor between these cases seems to be the timeline during which these patients developed the complications. Manna et al. reported in their case that patients developed subcutaneous emphysema on average 13.3 days following the onset of symptoms [2]. Staiano et al. reported an average of 10.75 days until the development of complications such as SPM [8]. In our case, the patient developed such complications approximately 10 days after the onset of her symptoms. Given these findings, one such strategy to prevent such complications could be to ensure proper evaluation of these patients within 10 days of symptom onset for signs of worsening disease. This could be especially important in the primary care setting, as this specialty frequently sees recently hospitalized patients.

## Conclusions

SPM is a complication of COVID-19 pneumonia that can be a prognostic indicator of worsening disease. The pathophysiology behind this complication seems multifactorial, and more studies are needed to determine the exact underlying cause. Treatment is dependent on the extent of the pneumomediastinum and usually consists of symptomatic control with close monitoring. This case adds to the growing literature on COVID-19-associated SPM in healthy patients with no history of ventilator use. Furthermore, we hope to highlight the importance of close follow-up in these patients to prevent and treat further complications and promote better outcomes.
